# Androgen receptor (AR) suppresses miRNA-145 to promote renal cell carcinoma (RCC) progression independent of VHL status

**DOI:** 10.18632/oncotarget.4522

**Published:** 2015-08-18

**Authors:** Yuan Chen, Yin Sun, Qun Rao, Hua Xu, Lei Li, Chawnshang Chang

**Affiliations:** ^1^ Sex Hormone Research Center, Department of Urology, Tongji Medical College/Hospital, Huazhong University of Science and Technology, Wuhan, China; ^2^ Department of Gynaecology and Obstetrics, Tongji Medical College/Hospital, Huazhong University of Science and Technology, Wuhan, China; ^3^ George Whipple Lab for Cancer Research, Departments of Pathology, Urology, and Radiation Oncology and Wilmot Cancer Center, University of Rochester Medical Center, Rochester, NY, USA; ^4^ Sex Hormone Research Center, China Medical University/Hospital, Taichung, Taiwan

**Keywords:** renal cell carcinoma, androgen receptor, microRNA-145, HIF2α

## Abstract

Mutational inactivation of the VHL tumor suppressor plays key roles in the development of renal cell carcinoma (RCC), and mutated VHL-mediated VEGF induction has become the main target for the current RCC therapy. Here we identified a signal pathway of VEGF induction by androgen receptor (AR)/miRNA-145 as a new target to suppress RCC progression. Mechanism dissection revealed that AR might function through binding to the androgen receptor element (ARE) located on the promoter region of miRNA-145 to suppress p53's ability to induce expression of miRNA-145 that normally suppresses expression of HIF2α/VEGF/MMP9/CCND1. Suppressing AR with AR-shRNA or introducing exogenous miRNA-145 mimic can attenuate RCC progression independent of VHL status. MiR-145 mimic in preclinical RCC orthotopic xenograft mouse model revealed its efficacy in suppression of RCC progression. These results together identified signals by AR-suppressed miRNA-145 as a key player in the RCC progression *via* regulating HIF2α/VEGF/MMP9/CCND1 expression levels. Blockade of the newly identified signal by AR inhibition or miRNA-145 mimics has promising therapeutic benefit to suppress RCC progression.

## INTRODUCTION

Renal cell carcinoma (RCC) is the most common type of kidney tumor and the most lethal urological cancer [19, 29], with high metastatic rate and poor prognosis in diagnosed RCC patients [21, 38]. To make matters worse, the metastatic RCC is highly resistant to most therapies including chemotherapy [4, 10]. Even though the recently developed therapy against VEGF signaling for advanced RCC has shown promising benefits in small groups of patients, the majority of advanced RCC patients remain refractory to the treatment [2, 16]. Thus, understanding the molecular mechanisms of RCC progression, specifically the metastasis, has strong significance to find a novel therapy to better battle the advanced RCC.

The epidemiological studies indicated that the gender difference with male: female ratio in RCC incidence is 1.6: 1.0 [8, 11], suggesting that sex hormones and their receptors may play important roles in the development of RCC. With crucial roles in many other diseases due to the gender difference [25, 26], AR's function in RCC metastasis is not clear. Early studies with clinical surveys found the AR could be detected at various stage of RCC, yet the linkage of AR expression to the RCC proliferation and metastasis remains unresolved [5, 15, 33].

MicroRNAs (miRNAs) can function as tumor suppressors or oncogenes in various cancers [36] and early studies suggested that miRNAs might play important roles in RCC progression [32]. However, the connection between miRNAs and AR-mediated RCC progression remains unclear. Here we identified a new signal showing AR-suppressed miRNA-145 played a key role to influence the RCC proliferation and invasion *via* upregulation of HIF2α/VEGF/MMP9/CCND1 signals.

## RESULTS

### AR promotes invasion and proliferation of various RCC cell lines

**Figure 1:**
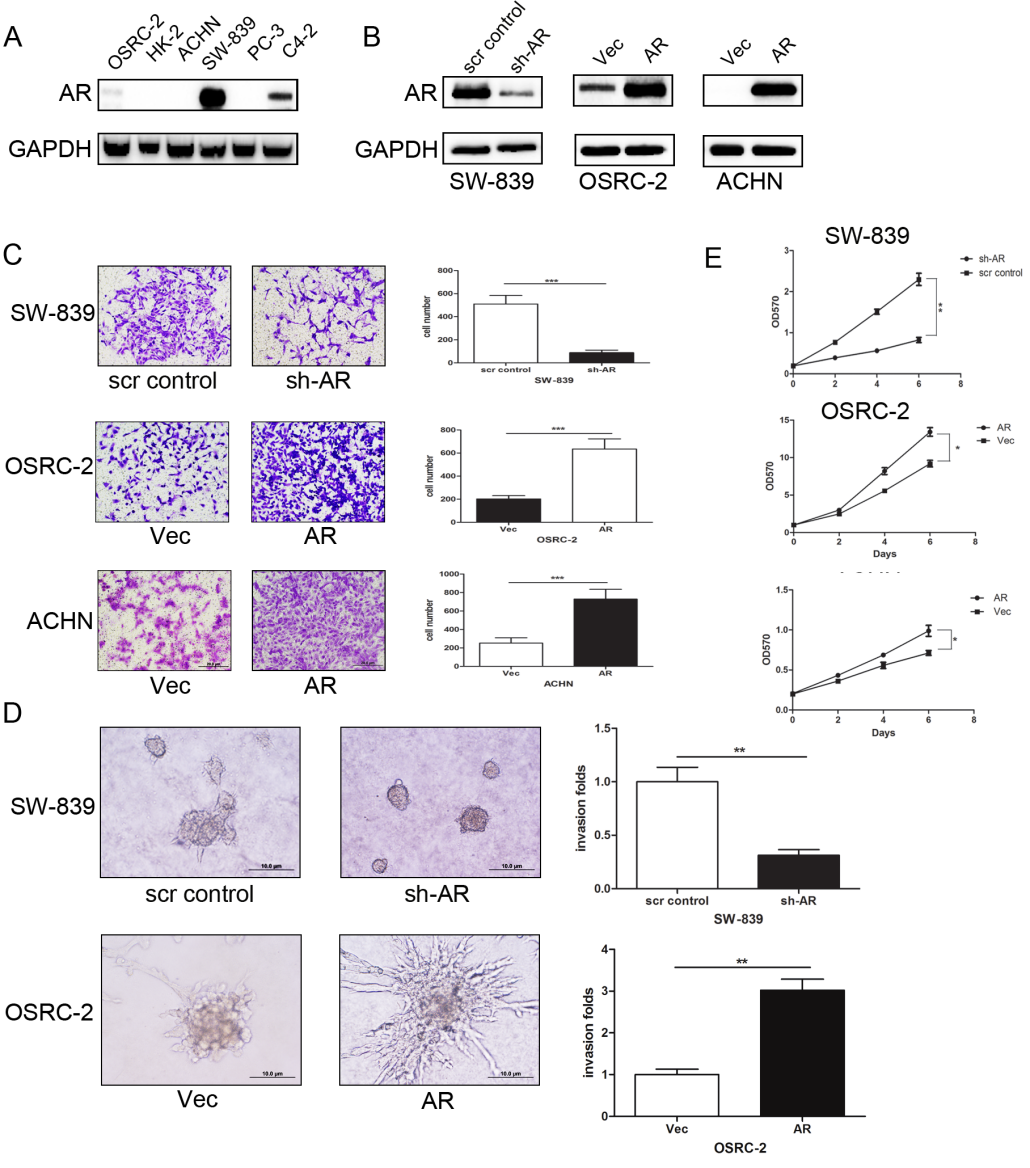
AR promotes RCC cell invasion and proliferation in various RCC cell lines **A.** Western blot analysis for the expression of AR in OSRC-2, HK2, ACHN and SW-839 cells. We used AR positive prostate cancer cell line C4-2 and AR negative prostate cancer cell line PC-3 as AR controls and GAPDH antibody staining was used as a loading control. **B.** The Western blot analysis for sh-AR in SW-839 cells compared with its scramble cells, over-expressed (OE) AR in OSRC-2 cells and ACHN cells compared with their vector cells with GAPDH antibody staining as a loading control. **C.** Invasion assay was performed using matrigel coated transwell chambers. sh-AR in SW-839 cells compared with its scr cells, OE AR in OSRC-2 cells and ACHN cells compared with their vector cells (sh-AR *vs*. scr control or AR *vs*.Vec). ***p < 0.001. **D.** RCC cells were grown on Matrigel for 10 days in 3D spheroid invasion assay. sh-AR in SW-839 cells compared with its scramble cells, OE AR in OSRC-2 cells compared with their vector cells (sh-AR *vs*. scr control or AR *vs.*Vec).**p < 0.01. **E.** Si-AR in SW-839 cells compared with its scramble cells, OE AR in OSRC-2 cells and ACHN cells compared with their vector cells (sh-AR *vs*. scr control or AR *vs.*Vec), anchorage-dependent cell growth was measured at the time points 2, 4, 6 days after lentivirus transfection using an MTT assay. *p < 0.05; **p < 0.01.

The VHL/HIF2α/VEGF signaling pathway has been used as therapeutic targets to suppress RCC progression since 2000 [12]. However, most of the therapies eventually fail due to the limited therapeutic efficacy or drug resistance. To find a new and better target, we focused on AR as its function has been linked well in other urological tumors, including prostate and bladder cancer [3, 7, 22, 28]. We first examined the AR expression in various RCC cell lines and found AR is highly expressed in SW-839 cells while has a lower expression in OSRC-2 and ACHN cells (Figure 1A). We then manipulated AR expression (Figure 1B) and explored its potential role in different RCC cells. Using matrigel-coated transwell invasion assay, we found knocking-down AR in SW-839 cells suppressed cell invasion as compared to SW-839 scramble controls (SW-839-scr control) (Figure 1C). In contrast, exogenous expression of AR in OSRC-2 cells (OSRC-2-AR) and ACHN cells (ACHN-AR) (Figure 1B) resulted in increased cell invasion compared with the control cells (Figure 1C).

Similar results were obtained when we replaced the matrigel-coated transwell invasion assay with another 3D culture invasion assay showing knockdown of AR led to drastically decreased cell invasion in SW-839 cells and overexpression of AR increased the invasion capacity in OSRC-2 cells (Figure 1D).

We also examined the effects of differential AR expression on RCC cell proliferation using MTT assays. The results revealed that knocking-down AR in SW-839 cells (SW-839-sh-AR) led to slower cell proliferation as compared to their scramble controls (SW-839-scr control) (Figure 1E). As expected, addition of AR in OSRC-2 cells (OSRC-2-AR) and ACHN cells (ACHN-AR) resulted in increased cell proliferation compared with the control cells (Figure 1E).

Taken together, results from Figure 1A-1E demonstrated that AR plays a positive role to promote RCC cell invasion and proliferation.

### AR suppresses miRNA-145 expression in RCC

**Figure 2:**
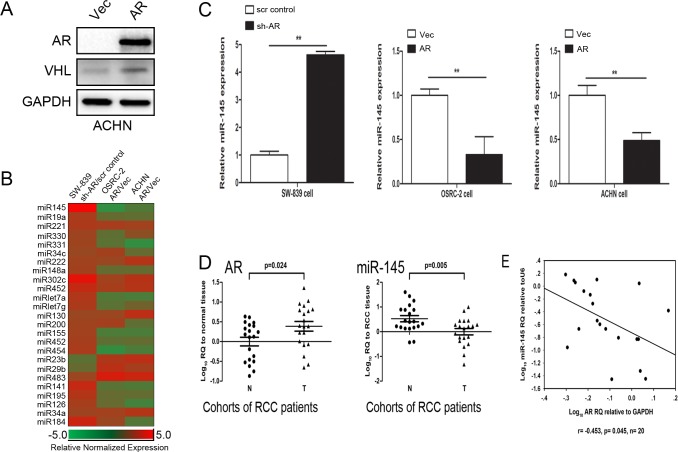
AR suppresses miR-145 expression in RCC cells **A.** Western blot analysis for AR, VHL of total lysates of VHL-wild-type ACHN cells with over-expressed AR and Vector (Vec), GAPDH antibody staining was used as a loading control. **B.** Real-time PCR of miRNAs related to RCC metastasis screened for sh-AR in SW-839 cells, over-expressed AR *vs.* Vector (Vec) in OSRC-2 cells and ACHN cells. **C.** Real-time PCR of miR-145 relative to U6 expression for sh-AR in SW-839 cells compared with its scramble (scr) cells, AR in OSRC-2 cells and ACHN cells compared with their vector (Vec) cells.**p < 0.01. **D.** Real-time PCR of AR and miR-145 expression in RCC tissues *vs.* adjacent normal renal tissues of 20 renal cancer patients. E: Plot of fold change of miR-145 related to fold change of AR for each patient; r, Pearson correlation coefficient.

To dissect the molecular mechanisms by which AR promotes RCC cell invasion and proliferation, we first examined its impact on VHL expression. Interestingly, we found addition of AR led to increase VHL expression in VHL wild-type RCC ACHN cells (Figure 2A), an opposite result to above Figure 1A-1E as increased expression of VHL, a tumor suppressor, should lead to suppress the RCC progression. These opposing results between Figure 1A-1E *vs.* Figure 2A implied that AR might function through alternative signals to promote the RCC progression.

We then focused on miRNAs as increasing evidence suggested that miRNAs might play profound roles in regulating tumor cell invasion and proliferation [13, 23]. We first applied an array of miRNAs that are related to RCC progression [35, 40] and found miRNA-145 was the most significantly suppressed by AR (Figure 2B-2C). To further confirm this finding, we altered the AR expression in various RCC cell lines and found that the miR-145 expression was consistently regulated by AR: knockdown of AR caused miR-145 induction in SW-839 cells and forced expression of AR in OSRC-2 and ACHN cells both led to reduction of miR-145 (Figure 2C).

To further strengthen the above conclusion, we then examined the AR *vs.* miR-145 expression in human RCC samples, and results revealed a significant negative correlation (r = −0.453) between the expressions of AR *vs.* miR-145 from 20 RCC samples (Figure 2D-2E).

Together, results from Figure 2B-2E suggested that AR could negatively regulate miR-145 expression in human RCC.

### AR enhances RCC cell invasion and proliferation *via* alteration of miR-145 expression

**Figure 3:**
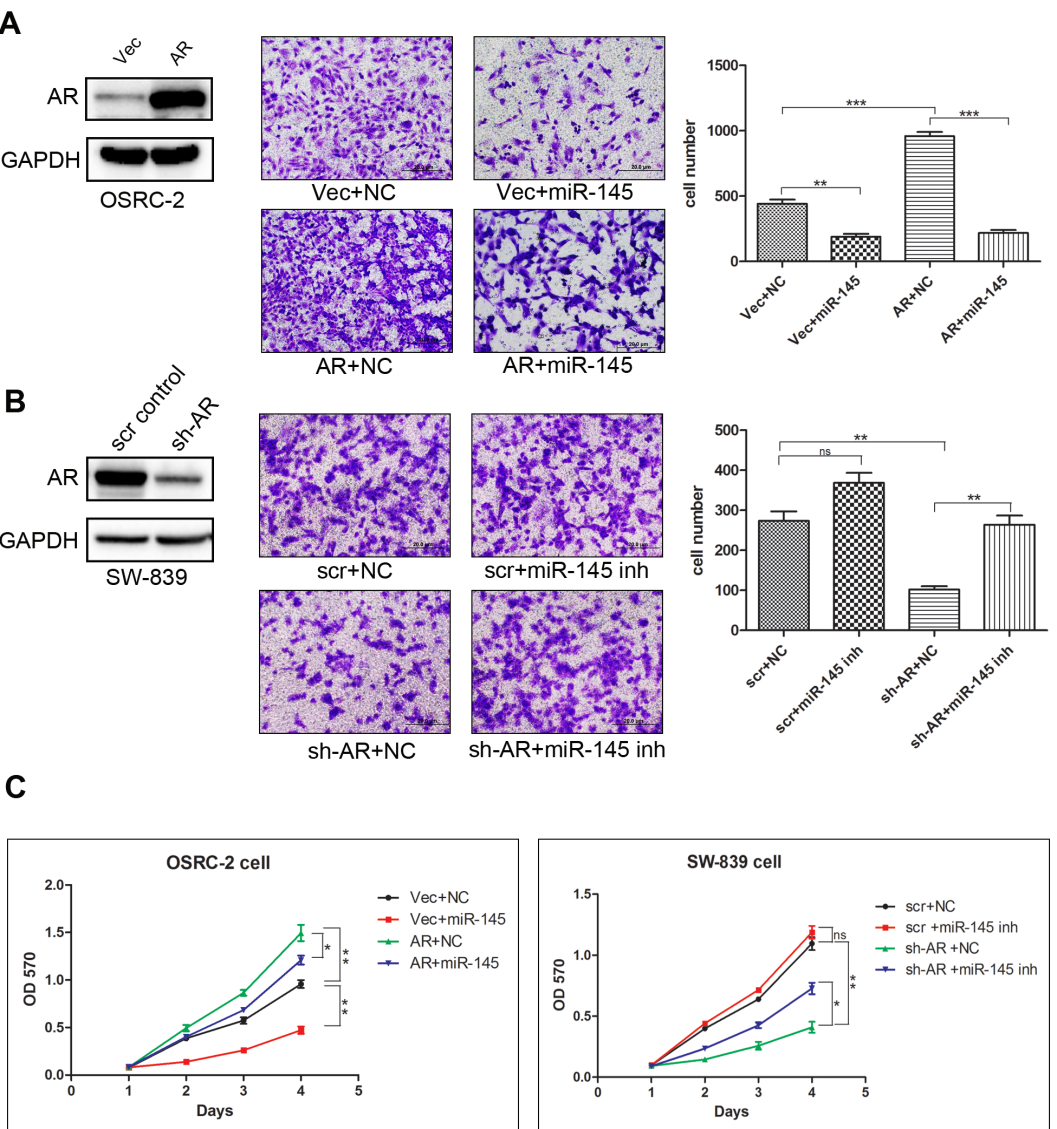
MiR-145 suppresses the invasion and proliferation of RCC regulated by AR **A.** Vector (Vec) or over-expressed (OE) AR OSRC-2 cell were transfected with negative control (NC) or miR-145 mimic. Invasion assay was performed using matrigel coated transwell chambers.**p < 0.01,***p < 0.001. **B.** Scr control or sh-AR SW-839 cells were transfected with NC RNA and miR145 inhibitor (inh). Invasion assay was performed using matrigel coated transwell chambers. **p < 0.01. **C.** Vector (Vec) or over-expressed AR OSRC-2 cell were transfected with NC or RNA and miR-145. Scr. control or sh-AR SW-839 cells were transfected with NC RNA or miR-145 inhibitor (inh). After 24 hours RNA transfection, anchorage-dependent cell growth was measured at 24, 48, 72 and 96 hours using an MTT assay. *p < 0.05; **p < 0.01.

To investigate whether miR-145 is involved in AR-enhanced RCC invasion, we applied interruption approaches and found that exogenous expression of miR-145 led to partially reverse the AR-enhanced RCC cell invasion (Figure 3A, AR+miR-145 *vs.* AR+NC, NC = negative control). Consistently, miR-145 inhibitor (inh) could partially reverse the negative impact of knocking-down AR on cell invasion (Figure 3B, sh-AR+miR-145 inh *vs.* sh-AR+NC).

A similar reciprocal relation with AR-enhanced RCC cell proliferation *via* modulation of miR-145 expression was also obtained in SW-839 and OSRC-2 cells (Figure 3C).

Together, results from Figure 3A-3C suggest that AR can function through downregulation of the miR-145 expression to enhance RCC cell invasion and proliferation.

### AR suppresses miR-145 promoter activity through regulation of p53

**Figure 4:**
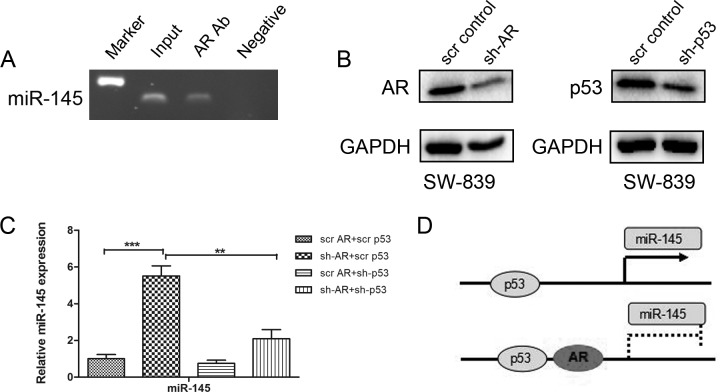
AR suppresses miR145 promoter activity through p53 **A.** Chromatin was prepared from SW-839 cells. Chromatin was incubated with AR antibodies or with Normal rabbit IgG as a negative control, DNA was extracted from immunoprecipitates and PCR-amplified using a pair of primers that flanked the AR DNA binding site in the promoter region of the miR-145. As a positive control, we also amplified the input chromatin DNA from control. **B.** The Western blot analysis for sh-AR and sh-p53 in SW-839 cells. GAPDH antibody staining was used as a loading control. **C.** QPCR results show the increased miR-145 in SW-839 cells after sh-AR can be partially reversed by sh-p53. **p < 0.01,***p < 0.001. **D.** Cartoon shows how AR suppresses miR-145 promoter activity through p53.

To further dissect the mechanism how AR modulates miR-145 at the molecular level, we applied ALLGGEN-PROMO virtual lab to predict the putative transcriptional factor binding sites on the miR-145 promoter region [17], and found that a 1.0-kb putative miR-145 promoter region might contain 4 potential AR response elements (AREs) located at 44, 446, 547 and 575 bp upstream to the transcriptional start site of the human pre-miR-145. We then performed the chromatin immunoprecipitation (ChIP) assays to determine whether AR might bind to the miR-145 promoter. The results in Figure 4A revealed that AR could bind to the miR-145 promoter *via* the ARE located at 575 bases upstream of the transcription start site, suggesting AR might modulate miR-145 expression at the transcriptional level.

Earlier studies suggested that under physiological conditions, p53 could also induce the miR-145 promoter activity *via* binding to the p53 response element (p53RE) [31]. We therefore examined whether AR might function through p53 to suppress miR-145 expression. As shown in Figure 4B and 4C, sh-AR-induced miR-145 expression was significantly attenuated when p53 was knocked-down by p53-shRNA.

Together, results from Figure 4A-4C suggest that that AR might suppress miR-145 expression *via* direct binding to the ARE located on the promoter region of miR-145 to suppress p53's induction of miR-145 (Figure 4D).

### AR-suppressed miR-145 regulates the HIF2α/VEGF/MMP9/CCND1 signals in the VHL mutant SW-839 and OSRC-2 cells

**Figure 5:**
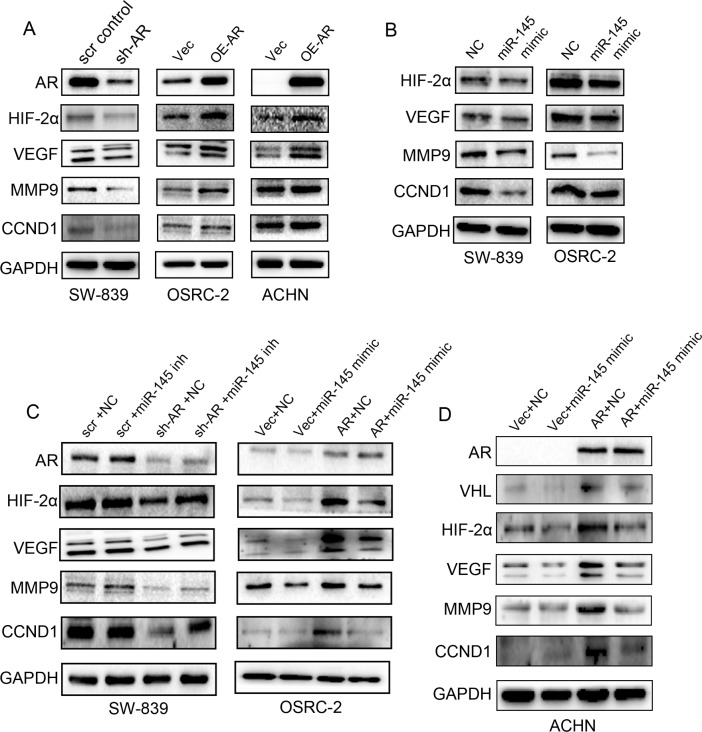
AR regulates the HIF2α expression via miR-145 **A.** Western blot analysis for AR, HIF2α, VEGF, CCND1 and MMP9 of total lysates of SW-839 cells with sh-AR and scr control; OSRC-2 and ACHN cells with over-expressed AR (OE-AR) and Vector (Vec) with GAPDH used to determine equal loading. **B.** Western blot analysis for AR, HIF2α, VEGF, CCND1 and MMP9 of total lysates of SW-839 and OSRC-2 cells transfected with negative control (NC) RNA or miR-145 mimic. GAPDH was used to determine equal loading. **C.** Western blot analysis shows the decreased HIF2α, VEGF, CCND1 and MMP9 in SW-839 cells after sh-AR can be partially reversed by miR145-inhibitor (inh). The increased HIF2α, VEGF, CCND1 and MMP9 in OSRC-2 cells with over-expressed AR can be partially reversed by miR-145 mimic. **D.** Western blot analysis shows the increased VHL, HIF2α, VEGF, CCND1 and MMP9 in ACHN cells after OE AR can be partially reversed by miR-145 mimic.

To further dissect the molecular mechanism(s) how AR-suppressed miR-145 signals may influence the RCC progression, we first applied focus array to those reported metastasis-related genes that are linked to RCC progression [1]. We paid special attention to the HIF2α*/*VEGF signals since these two downstream targets of VHL have been reported to promote cancer progression *via* promoting angiogenesis [14] and were two of the essential targets for the current therapies for advanced RCC [27]. The results revealed that knocking-down AR in SW-839 cells (SW-839-sh-AR) suppressed the expression of HIF2α and its downstream targets including VEGF, MMP9 and CCND1 compared to their scramble controls (SW839-scr control) (Figure 5A). In contrast, addition of functional AR in OSRC-2 cells (OSRC-2-AR) and ACHN cells (ACHN-AR) resulted in increased expression of HIF2α, VEGF, MMP9 and CCND1 compared with their vector (Vec) control cells (Figure 5A).

During our studies of miR-145 on regulation of HIF2α/VEGF, a report confirmed our findings that in glioblastoma cells miR-145 can directly silence HIF2α expression through targeting the 3′UTR of HIF2α [43]. We then extended these studies and demonstrated that adding miRNA-145 mimic also led to suppress the expression of HIF2α, VEGF, MMP9 and CCND1 in SW-839 and OSRC-2 cells (Figure 5B), and importantly, the interruption approach using miR-145 inhibitor (inh) or miR-145 mimic could partially reverse the AR effect (either *via* suppressing AR or enhancing AR) on the expression of HIF2α, VEGF, MMP9 and CCND1 (Figure 5C, sh-AR+miR-145 inh *vs.* sh-AR+NC) vs (Figure 5C, AR+miR-145 mimic *vs.* AR+NC).

Together, results from Figure 5A-5C suggest that AR suppressed miR-145 expression to promote RCC progression *via* enhancing the HIF2α/VEGF/MMP9/CCND1 signals in the VHL mutant SW-839 and OSRC-2 cells.

### AR-suppressed miR-145 signals can override the AR-enhanced VHL signals in VHL wild-type ACHN cells

As HIF2α/VEGF signals are also the key downstream targets of VHL, we examined the miR-145 impact on VHL and found miR-145 alone could suppress VHL expression in ACHN cells with wild-type VHL (Figure 5D). Importantly, addition of miR-145 could reverse the AR-enhanced VHL expression (Figure 5D) with a consequent increase of downstream signals. These results suggest that AR-suppressed miR-145 signals can override the opposing AR-enhanced VHL to result in the increase of the HIF2α/VEGF (as well as MMP9 and CCND1) signals in VHL wild-type RCC ACHN cells (Figure 5D).

Together, results from Fig.5A-5D demonstrated that AR suppressed miR-145 signals might play key roles to influence the RCC progression *via* boosting the HIF2α/VEGF/MMP9/CCND1 signals in either VHL-wild-type or VHL-mutant cells.

### AR enhanced RCC growth and metastasis *via* suppression of miR-145 thus increasing HIF2α in RCC *in vivo*

**Figure 6:**
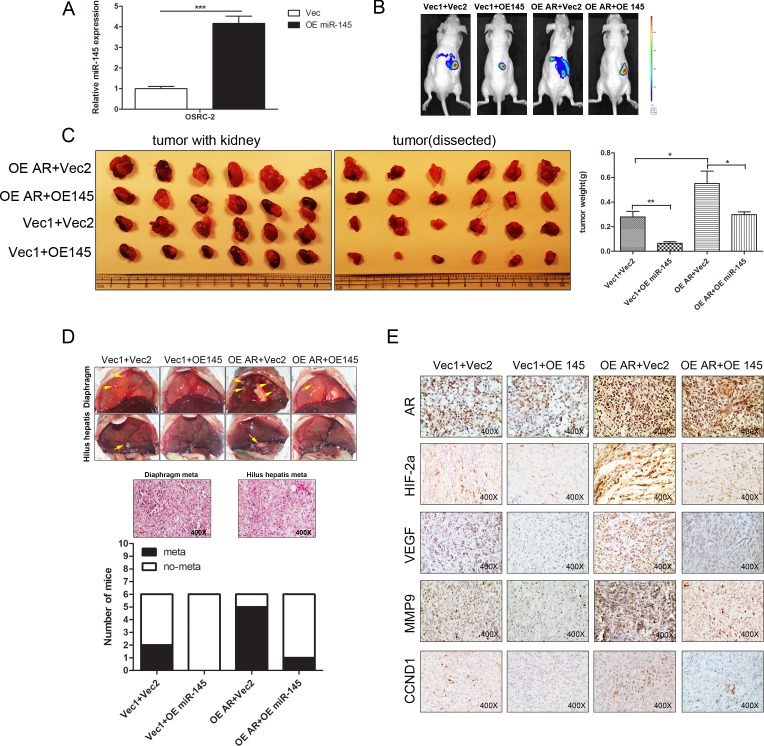
AR promotes growth and metastasis of RCC *via* suppressing miR-145 *in vivo* **A.** Construction and confirmtion of OSRC-2 cells with stable high miR-145 expression using QPCR. **B.** The 1×10^6^ OSRC-2 cells with over-expressed AR and Vec2 (OE AR+vec2), Vector AR (Vec1+Vec2), OE AR plus stable high miR-145 expression (OE AR+OE 145), Vector AR plus stable high miR-145 expression (Vec1+OE 145) orthotopically implanted into the kidney of nude mice. 5 weeks after implantation, the RCC growth and metastases were monitored by IVIS images. **C.** Then the mice were sacrificed and the primary tumor sizes in each group were observed (left picture: tumors with kidney, right picture: tumors only) and weighed. **D.** The metastatic tumors in the diaphragm and hepatic hilar region were observed and confirmed by histology H&E staining after sacrifice. E. IHC staining confirmed the expression of AR, HIF2α, VEGF, MMP9 and CCND1 in different groups.

To prove the above *in vitro* data *in vivo*, we first generated the miRNA-145-expressing OSRC-2 cells (Figure 6A) and then orthotopically xenografted these OSRC-2 cells (OE AR+OE miR-145; OE AR+NC; Vec-AR+OE miR-145 and Vec-AR+NC) under the nude mice kidney capsules (1×10^6^ cells) with IVIS Imaging system to monitor tumor progression. The results (after 5 weeks) revealed that addition of AR in OSRC-2 cells increased growth and tumor weight in athymic nude mice compared to Vec-AR OSRC-2 cells, while stable expression of miR-145 in the OE-AR OSRC-2 cells could partially reverse the positive impact of AR and decreased the RCC tumor growth and tumor weight (Figure 6B-6C).

We also found (from IVIS image) increased metastatic foci in 5 out of 6 mice with AR-over-expressed OSRC-2 cells. In contrast, 2 out of 6 mice showed such metastatic foci in the control cells. Importantly, only 1 out of 6 mice showed the metastatic spot with simultaneous OE-miR-145 and OE-AR implantation, while there was no metastasis in cells with OE-miR-145 implants only (Figure 6B). Furthermore, H&E staining of those metastatic foci confirmed that those are indeed RCC cells, including those in diaphragm and hepatic hilar region (Figure 6D), and IHC staining for AR, HIF2α, VEGF, MMP9 and CCND1 were also in agreement with *in vitro* results showing AR-suppressed miR-145 is a key player to suppress RCC progression *via* modulation of HIF2α/VEGF/MMP9/CCND1 signals (Figure 6E).

## DISCUSSION

Few studies linked the miRNAs to AR function in RCC progression even though recent reports demonstrated that some miRNAs might play a role in AR-mediated signals in prostate cancer progression [18, 20, 30, 34]. For example, Shi *et al* found AR could function through up-regulating the miR-125b expression to suppress Bak1 expression to promote prostate cancer progression [34]. Ribas *et al* also found that AR could directly bind to miR-21 promoter to exert its influence on the prostate cancer growth [30], and Murata *et al* reported that miR-148a was an androgen-responsive miRNA that could promote prostate LNCaP cell growth *via* repressing its target CAND1 expression [20]. However, few studies linked miRNA-145 to AR function in RCC progression even though its important in other tumors [9, 24, 39].

**Figure 7:**
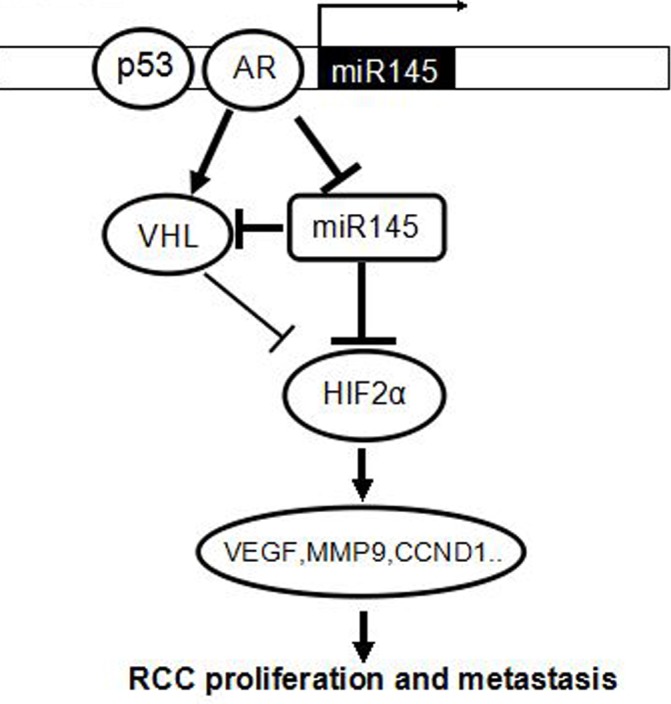
A schematic model of the relationship among AR, miR-145,VHL and HIF2α

Our results showing AR-suppressed miR-145 could influence RCC progression represents the first evidence to link the miR-145 regulation to the AR function in RCC. More importantly, in the RCC cells with wild-type VHL, our results revealed that wild-type VHL expression was also enhanced after exogenous AR expression in ACHN cells, and this increased wild-type VHL expression could be suppressed by addition of miR-145. The exact mechanism and biological significance of this AR-induced VHL remains to be determined. Nevertheless, it is clear that AR induces HIF2α/VEGF signals irrespective of VHL status, particularly when it can increase VHL as shown in Figure 5D, thus was expected to reduce HIF2α, yet it resulted in HIF2α elevation. This explains well why AR-induced VHL may still fail to promote RCC progression and suggests a dominant role of AR's effect on HIF2α/VEGF regulation (Figure 7). These findings imply that VHL is an initiating genetic change for RCC, but not the only determinant for the malignancy of RCC. In addition, our data have strong clinical implication since current therapies designed to target the induced HIF2α/VEGF signals due to mutant or suppressed VHL (*via* promoter hyper-methylation) only yield limited efficacy in select RCC patients [6]. Therefore, the development new therapies against AR either with AR-siRNAs or the newly developed AR degradation enhancer ASC-J9^®^ [37, 41, 42] or introducing miR-145 mimic shall provide an alternative therapeutic option to better suppress the induced HIF2α/VEGF signals.

Another potential advantage of targeting AR-suppressed miR-145 signals may come from the profound AR effects to suppress RCC progression that suppression of HIF2α-VEGF signal alone may not be able to have, and early studies using ASC-J9^®^ to suppress AR-mediated prostate cancer/bladder cancer progression all resulted in reasonable efficacy with little side effects in tested mice [37].

In summary, these results identified the AR-suppressed miR-145 as a key player to influence the RCC progression *via* promoting the HIF2α/VEGF/MMP9/CCND1 signals. Targeting these newly identified signals including AR and/or miR-145 with small molecules may help us to better suppress RCC progression and invasion.

## MATERIALS AND METHODS

### Cell culture and transfection

The human renal cell carcinoma (RCC) cell lines SW-839, ACHN and the normal primary renal tubular cell line HK2, were purchased from American Type Culture Collection (ATCC, Manassas, VA), RCC cell line OSRC-2 was purchased from the Riken Cell Bank (RCB, Tsukuba, Japan). The SW-839, OSRC-2, ACHN cells and HK2 cells were cultured in Dulbecco's Modified Eagle Medium (DMEM) supplemented with 10% fetal bovine serum (FBS) in humidified 5% CO_2_ environment at 37°C.

For lentivirus-based transfection, viruses were generated using psAX2/pMD2G system. Briefly, psAX2, pMD2G with pWPI-based cDNA or pLKO1-based shRNA (1:1:2) were co-transfected into 293T cells. After 48 hours, virus supernatants were collected and stored in −80°C for future use. AR siRNA and p53 siRNA were transfected into RCC cells and selected with 1μg/μl puromycin. For transient transfection, miR-145 mimic (10 nM) and miR-145 inhibitor (10 nM; both Qiagen) with their corresponding negative controls were transfected using the Lipofect AMINE 2000 (Invitrogen) following the manufacturer's instructions.

### Real-time quantitative PCR assays

Total RNAs were extracted using Trizol reagent (Invitrogen, Grand Island, NY). MiRNAs were isolated by using PureLink^®^ miRNA kit. In brief, 50 ng small RNAs were processed for poly A addition by adding 1 unit of polymerase with 1 mM ATP in 1xRT buffer at 37°C for 10 minutes in 10 μl volume, heat inactivated at 95°C for 2 minutes, adding 50 pmol anchor primer to 12.5 μl, and incubated at 65°C for 5 minutes. For the last step of cDNA synthesis, add 2 μl 5x RT buffer, 2 μl 10 mM dNTP, 1 μl reverse transcriptase to total of 20 μl, and incubate at 42°C for 1 hour [25]. Quantitative real-time PCR was conducted using a Bio-Rad CFX96 system with FAM/FITC to determine the miRNA expression level, expression levels, which were normalized to the expression of U6 RNA. To test the the mRNA expression level of interest gene, reverse transcription was performed using Superscript III transcriptase (Invitrogen, Grand Island, NY). Quantitative real-time PCR (qRT-PCR) assays were performed using a Bio-Rad CFX96 system with SYBR green to determine the mRNA expression level expression levels that were normalized to the expression of GAPDH RNA.

### Western blot analysis

Cells were lysed in RIPA buffer and proteins (20 μg) were separated on 8–10% SDS/PAGE gel and then transferred onto PVDF membranes (Millipore, Billerica, MA). After blocking membranes, they were incubated with appropriate dilutions of specific primary antibodies. The following primary antibodies were used: rabbit anti-AR (1:1000; Santa Cruz Biotechnology, CA); mouse anti-p53 (1:1000; Cell Signaling, MA); rabbit anti-HIF2α (1:1000; Abcam, Cambridge, MA); rabbit anti-VEGF (1:1000; Abcam); rabbit anti-MMP9 (1:1000; Abcam); mouse anti-CCND1(1:1000; Cell Signaling) and mouse anti-GAPDH (1:1000; Santa Cruz Biotechnology). The blots were incubated with HRP-conjugated secondary antibodies and visualized using the ECL system (Thermo Fisher Scientific, Rochester, NY).

### RCC cell invasion assay

The invasive capability of RCC cells was determined by matrigel-coated transwell assay. Before seeding the cells, Matrigel (BD, Inc.) was diluted 5 fold with serum-free DMEM medium, and the dilution was loaded and dried in the upper chambers of 8.0 μm-pore-size polycarbonate membrane filters (Corning Inc., Corning, NY). 1×10^5^ harvested RCC cells were seeded on the upper chamber in serum-free DMEM and DMEM with 10% FBS was used as attractant in the lower chamber. Cells were allowed to migrate for 48 hours and then invaded cells attached to the lower surface of the membrane were fixed by 4% paraformaldehyde and stained with 0.1% toluidine blue. Cell numbers were counted in five randomly chosen microscopic fields (200×) per membrane.

### 3D invasion assay

40 μl thawed Matrigel was evenly loaded to each well of 8-well glass chamber slide (at 50 μl/cm^2^). After Matrigel solidification, 1×10^4^ SW-839 and OSRC-2 cells with manipulated AR were added with media containing 5% Matrigel and 10 ng/ml EGF. Media was changed every 4 days with assay media containing 2.5% Matrigel and 5.0 ng/ml EGF. After 10 days incubation, cell structures were captured in 10 different random fields under 200× microscope.

### RCC cell proliferation assay

RCC cells were seeded in 24-well plates (3000 cells/well), then cell numbers were quantified at different time points (0, 2, 4, and 6 days) by incubating cells in 0.5 mg/ml of 3-(4,5-dimethylthiazol-2-yl)-2,5-diphenyltetrazolium bromide (MTT) (Sigma-Aldrich) for 1 hour and dissolved in DMSO, followed by recording absorbance at 570nm.

### Chromatin immunoprecipitation assay (ChIP)

Cell lysates were pre-cleared sequentially with normal rabbit IgG (sc-2027, Santa Cruz Biotechnology) and protein A-agarose. Anti-AR antibody (2.0 μg) was added to the cell lysates and incubated at 4°C overnight to prepare chromatin for assays. For the negative control, IgG was used in the reaction. Speciﬁc primer sets designed to amplify a target sequence within human miR-145 promoter were: human miR-145 precursor; Forward 5′-cttgtgatgctggggaagtt-3′; Reverse, 5′-tgggctcagaaagagaaagc-3′; PCR products were identified by agarose gel electrophoresis.

### Generate RCC cell lines with stable expression of miR-145

The miRNA-145 expression plasmid was generated by cloning the genomic pre-miR-145 gene, with a 300-bp sequence on each flanking side, into retroviral transfer plasmid pMSCV-puro to generate plasmid pMSCV-miR-145. pMSCV-miR-145 was co-transfected with the pIK packaging plasmid in 293T cells using the standard calcium phosphate transfection method. After 36 hours co-transfection, supernatants were collected and incubated with cells to be infected for 24 hours in the presence of polybrene (8 μg/ml). After infection, puromycin (1.0 μg/μl) was used to select stably transduced cells.

### Patient specimens

Tumor specimens and adjacent normal kidney tissues were collected from a total of 20 patients undergoing nephrectomy for RCC at the Huazhong University of Science and Technology affiliated Tongji Medical Hospital. All specimens were obtained on the basis of their availability for research purposes and under a protocol approved by the local medical ethics committee of Tongji Medical Hospital. Written consent was obtained from the patients prior to the study.

### *In vivo* metastatic studies

Male 6-8 weeks old nude mice were purchased from NCI [24]. For types of different engineered OSRC-2 cells (Vector control; AR-expressing; Vector plus miR145; and AR plus miR145) were injected into mice renal capsule mixed with equal amounts of Matrigel. After 5 weeks, metastasis were determined using a Fluorescent Imager (IVIS Spectrum, Caliper Life Sciences, Hopkinton, MA). Then mice were sacrificed and tumors/metastases removed for H&E and IHC staining.

### HE staining

After dewaxing and rehydration, the paraffin embedded and sliced sections were stained in haematoxylin for 5 min and washed in running tap water for 5 min. Then the sections were stained in eosin for 30 s, dehydrated, and mounted by routine methods.

### Immunohistochemistry

The fresh kidney tumors of mice were fixed in 4% para-formaldehyde and embedded in paraffin, then sectioned at a thickness of 5 μm. After dewaxing and rehydration, sections were treated with 10% H_2_O_2._ After 5% BSA blocking, all sections were incubated with the primary antibodies at 4°C overnight. The primary antibody was recognized by the biotinylated secondary antibody (Vector), and visualized by VECTASTAIN ABC peroxidase system and peroxidase substrate DAB kit (Vector). The primary antibodies used were: rabbit anti-AR (Santa Cruz), rabbit anti-MMP9 (Abcam), mouse anti-HIF2α (Abcam), rabbit anti-VEGF (Abcam) and rabbit anti-CCND1 (Cell Signaling).

### Statistical analysis

Data are expressed as mean±SEM from at least 3 independent experiments. Statistical analyses involved paired *t* test with SPSS 17.0 (SPSS Inc., Chicago, IL). *For in vivo* studies, measurements of tumor metastasis among the four groups were analyzed through one-way ANOVA coupled with the Newman-Keuls test. *P* < 0.05 was considered statistically significant.
